# Current understanding of gliomagenesis: from model to mechanism

**DOI:** 10.7150/ijms.77287

**Published:** 2022-11-14

**Authors:** Juanjuan Luo, Muhammad Junaid, Naima Hamid, Jin-Jing Duan, Xiaojun Yang, De-Sheng Pei

**Affiliations:** 1 School of Public Health and Management, Chongqing Medical University, Chongqing 400016, China.; 2 Guangdong Provincial Key Laboratory of Infectious Disease and Molecular Immunopathology, Shantou University Medical College, Shantou 515041, China.; 3 University of Chinese Academy of Sciences, Beijing 100049, China.

**Keywords:** Glioma, Tumorigenesis, Signaling pathways, CRISPR/Cas9, Model

## Abstract

Glioma, a kind of central nervous system (CNS) tumor, is hard to cure and accounts for 32% of all CNS tumors. Establishing a stable glioma model is critically important to investigate the underlying molecular mechanisms involved in tumorigenesis and tumor progression. Various core signaling pathways have been identified in gliomagenesis, such as RTK/RAS/PI3K, TP53, and RB1. Traditional methods of establishing glioma animal models have included chemical induction, xenotransplantation, and genetic modifications (RCAS/t-va system, Cre-loxP, and TALENs). Recently, CRISPR/Cas9 has emerged as an efficient gene editing tool with high germline transmission and has extended the scope of stable and efficient glioma models that can be generated. Therefore, this review will highlight the documented evidence about the molecular characteristics, critical genetic markers, and signaling pathways responsible for gliomagenesis and progression. Moreover, methods of establishing glioma models using gene editing techniques and therapeutic aspects will be discussed. Finally, the prospect of applying gene editing in glioma by using CRISPR/Cas9 strategy and future research directions to establish a stable glioma model are also included in this review. In-depth knowledge of glioma signaling pathways and use of CRISPR/Cas9 can greatly assist in the development of a stable, efficient, and spontaneous glioma model, which can ultimately improve the effectiveness of therapeutic responses and cure glioma patients.

## Introduction

Glioma is a common human brain tumor that accounts for approximately 50% of intracranial tumors. Although the current incidence of center nervous system (CNS) tumors is 14.3% of all tumors, 49.1% of nervous system tumors are malignant gliomas 1. Advanced gliomas, which are also named primary glioblastoma (GBM), are considered one of the deadliest malignant tumors in the world 2. Glioma incidence varies by age; for example, common central nervous system (CNS) tumors in children include pilocytic astrocytomas and embryonal tumors, whereas common CNS tumors in adults include meningiomas, pituitary tumors, and malignant gliomas 3. In addition to age differences, glioma incidence varies by sex, ethnicity, tumor histology, and tumor subtype. Besides, the CNS microenvironment is naturally equipped to control proliferative cells and lead to cancer development 4-6. Recently, a new classification system for glioma tumors by integrating both tumor morphology and biomolecular features 7. The classification evaluated the correlation between microarray expression profiling of gliomas and tumor grades, progression, malignancy, and prognosis to identify the molecular subtypes 8. A recent study indicated that the characteristics of gene expression in the gliomas derived from transgenic zebrafish supported the notion that different molecular mutation-selective pressures drive different progression of gliomagenesis 9.

In recent decades, the survival rate of glioma patients has remained low due to poor prognosis, with less than 5% five-year survival rate, and the prognosis is even worse in elderly patients with glioma 10. Drug-based curative measures are not ideal for treating glioma because of insufficient understanding of the molecular mechanisms associated with gliomagenesis 11, 12. In addition, the limited and vague identification of early-stage gliomas also leads to delays in glioma treatment. Recently, genetic markers were proven to be effective clinical signs that can be measured with precision to predict glioma outcome 13. To date, three core signaling pathways have been identified for high-grade glioma, namely, the receptor tyrosine kinase (RTK)/phosphatidylinositol 3'-kinase (PI3K)/alpha serine-threonine protein kinase (AKT), phosphoprotein53 (TP53), and retinoblastoma (RB1) signaling pathways 14-16. Therefore, it is necessary to modify the current techniques and establish new methods to improve the prognosis of glioma patients.

Cancer cells have the fundamental features of growth and survival beyond a nominal homeostatic or favorable environment, and their growth, survival, oncogenic proliferation, and apoptosis are governed by signaling pathways 12. Several methods of genetic modification, including RCAS/t-va, Cre-loxP, zinc finger nucleases (ZFNs), and Tal-effector nuclease (TALENs), have introduced a new dimension in the development of genome manipulation at the molecular level 17-20. However, these gene editing techniques have various limitations, such as high cost, difficult design, labor intensiveness, low efficiency, and time consumption 21. Specifically, CRISPR/Cas9 system, a powerful gene-editing tool, includes a guide RNA (gRNA) and programmable nuclease (Cas9) that can process double-strand breaks (DSBs) at specific target sites efficiently and accurately via homology-directed repair (HDR) or nonhomologous end joining (NHEJ) 22, 23. Therefore, it is essential to highlight the benefits of developing a new glioma model using CRISPR/Cas9 to better understand the molecular mechanisms of gliomagenesis and establish better therapeutic methods.

**Figure 1:**
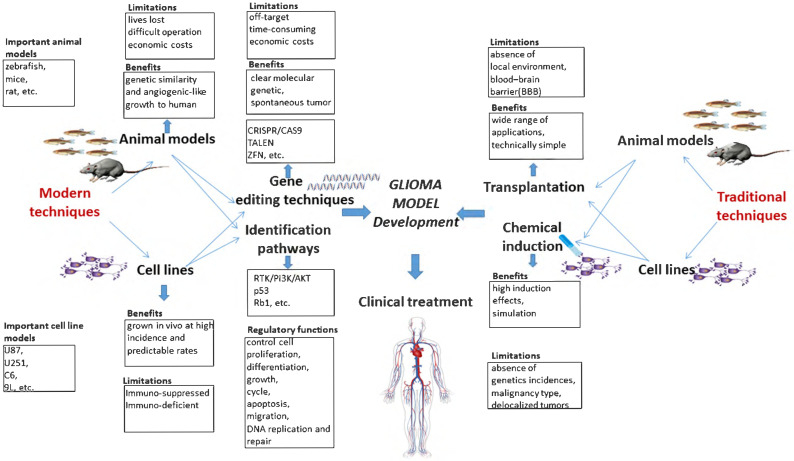
Schematic diagram highlighting the pros and cons of traditional and modern glioma models, gene editing techniques and key pathways and their regulatory functions.

Here, we presented a systematic view of the molecular characteristics of gliomagenesis in the context of genetic markers and core signaling pathways. Furthermore, comparisons are drawn among traditional and modern methods for establishing glioma models. Moreover, the advantages and limitations of traditional and modern gene editing techniques are critically discussed. The potential applications of CRISPR/Cas9 system for generating glioma models are specifically highlighted, and the clinical trials associated with gene therapy are briefly discussed. Finally, clinical trials and available glioma therapies are also briefly discussed in the current study. A summary highlighting the pros and cons of traditional and modern glioma models, gene editing techniques, key signaling pathways and their regulatory function is presented in the form of a schematic diagram (Fig. 1).

## Overview of the molecular classification of gliomas

**Figure 2:**
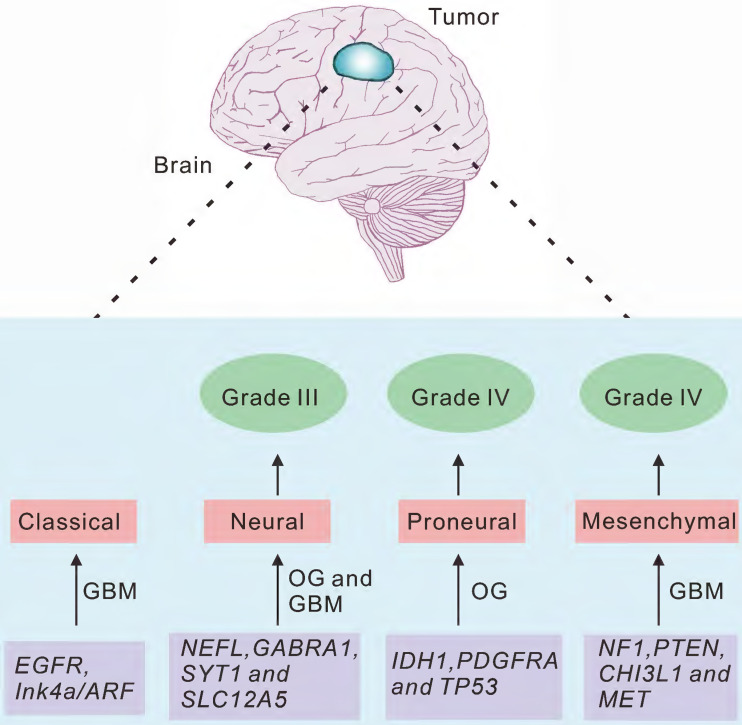
Characteristics of the molecular classification of gliomas.

Previous studies indicated that the new molecular classification system for gliomas might be valuable for predicting the prognostic of the glioma patients 24, 25. We therefore summarized the molecular signatures associated with tumor aggressiveness and progression, as well as the correlation between these signatures and the signaling pathways implicated in gliomagenesis 8, 17. Several subtypes, including classical, neural, proneural and mesenchymal subtypes, were named based on the expression of signature genes (Fig. 2).

### Classical subtype

Chromosome 10 (Chr 10) loss combined with chromosome 7 (Chr 7) amplification was detected in all classical subtype, and frequently occurred in GBM patients. Although the Chr 7 amplification was also observed in other subtypes of glioma, the highly expression of EGFR was infrequently determined in other subtypes. It is known that *TP53* mutation is one of the most frequently event in GBM patients. However, compared with the highly frequency of *EGFR* mutation, *TP53* mutation is rarely observed in classical subtype 14. In addition, homozygous deletion of 9p21.3, which targets *CDKN2A*, was a frequent and significantly associated event in classical subtype, and accompanied with EGFR amplification in most of classical subtype samples. Furthermore, previous report showed that the neural precursor and stem cell marker NES, and the regulators of Notch and Sonic hedgehog signaling pathways, including NOTCH3, JAG1, LFNG, SMO, GAS1 and GLI2, were frequently amplified in classical subtype 16.

### Neural subtype

The neural subtype was identified by the amplification of several neuron markers, including SYT1, GABRA1, NEFL and SLC12A5, which were involved in the processes of neuron projection and axon and synaptic transmission 16. In addition, extensive pathological analysis confirmed the diagnosis of GBM events in samples of this subtype 16. This classification offers a markedly better prognosis and includes the expression of certain genes, which were involved in the process of neurogenesis in the normal brain 8.

### Proneural subtype

The mutations of *PDGFRA* and *IDH1* were two major features in proneural subtype. Although PDGFRA was amplified in almost all subtypes of GBM, it was amplified with a much higher rate in proneural subtype 8. However, the characteristic signature of PDGFRA in proneural samples is accompanied by focal amplification and highly expression of *PDGFRA* gene 16, 26. In addition, ten out of sixteen *PIK3CA/PIK3R1* mutations were observed in proneural subtype, most of which did not have PDGFRA abnormalities, whereas eleven out of twelve mutations of *IDH1* gene were determined in this subtype with no* PDGFRA* abnormality 16. It is noted that the mutations of *TP53* were also frequently detected in this subtype. In contrast, the classical GBM event, the amplification of Chr 7 paired with the loss of Chr 10, were distinctly less prevalent and only occurred in 54% proneural subtype samples. The highly expressed oligodendrocyte development genes, including *PDGFRA, NKX2-2* and *OLIG2*, were detected in proneural subtype 27. Notably, the amplification of *OLIG2* could promote proliferation and induce tumorigenesis through downregulating CDKN1A expression 28.

### Mesenchymal subtype

The mesenchymal subtype mainly exhibited the amplification of the mesenchymal markers, including *CHI3L1* and *MET* 8. Focal hemizygous deletions of NF1 predominantly occurred in the mesenchymal subtype 16. Verhaak et al. reported that, 70% samples were defined as mesenchymal subtype in *NF1* mutated samples. In addition, most of mesenchymal subtype samples were detected as *NF1* and *PTEN* dual mutations, which play an important role in the RTK/PI3K pathway 16.

## Critical genetic markers and signaling pathways in gliomagenesis

With the continuous development of sequencing technologies and high-throughput gene editing, it is possible to analyze the genetic and epigenetic changes in tumors 29. In 2009, The Cancer Genome Atlas Network (TCGA) analyzed the variations in 601 types of tumor-associated genes by utilizing gene sequencing in more than 200 glioma samples 14. TCGA analysis of DNA methylation, DNA copy number and other genetic mutations in glioma patients revealed the core glioma pathways, and their regulatory functions were summarized in the RTK/PI3K/AKT, TP53 and RB1 signaling pathways. Chow et al. found gene mutations (in *Pten*, *Tp53* and *Rb1*) in mice that induced high-degree malignant tumors in astrocytes 26. They showed that the cooperation within the aberrant Pten, Tp53, and Rb1 pathways can induce high-grade astrocytoma in the mouse brain. In this context, NF1 and PTEN, which are both involved in PI3K/AKT pathway, abrogated the major negative regulator restraining PI3K activation. RB1 regulated cell cycle, and TP53 is a tumor suppressor that regulates cell death. In addition, in glioma patients, several studies have indicated that at least one aberrant pathway among the RTK/PI3K/AKT, TP53 and RB1 signaling pathways was identified in 80-90% of glioblastomas 30, 31, which highlights their potential as therapeutic targets.

### RTK/PI3K/AKT pathway

**Figure 3:**
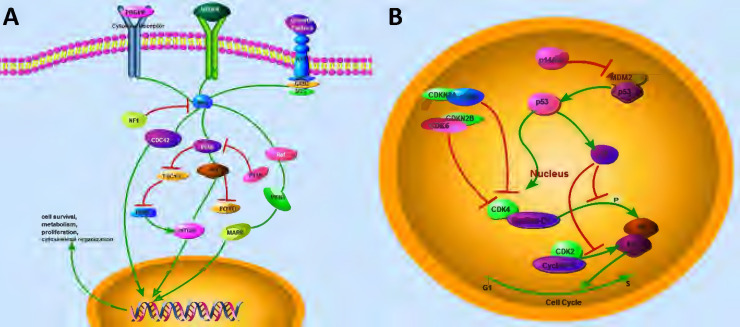
** Critical genetic markers and signaling pathways in gliomagenesis. (A)** The growth-factor-RTK signaling pathway. The ligands of RTKs, including PDGFR and EGFR, activate three downstream cascades in glioma. RAF/MEK/MAPK, PI3K/AKT, and CDC42 pathways are responsible for cell survival, metabolism, proliferation, and cytoskeletal organization, respectively. **(B)** TP53 and RB1 pathways. *MDM2* gene contains a TP53 site, which can bind to TP53 and abolish its transcriptional activity. P14ARF could interact directly with MDM2, thereby resulting in the stabilization of both TP53 and MDM2. TP53 can promote the transcription of p21, which is capable of silencing CDKs. CDK4/6 and CDK2 can bind to D-type cyclins and E-type cyclins, respectively, and induce the release of E2F transcription factor, which activates the expression of a set of genes critical for regulating G_1_/S transition. CDKN2A and CDKN2B bind to CDK4/6, respectively, and inhibit CDK4/cyclin D1 complex, resulting in the inactivation of RB1-mediated G_1_/S transition.

In RTK/Ras/PI3K pathway, receptor tyrosine kinases (RTKs) can mediate many growth signals through diffusive growth factors (Fig. 3A). Several ligands of RTK, including PDGFR, EGFR and VEGFR 32, are frequently overexpressed in GBM specimens 33. In this context, the amplification of EGFR, which is the most common RTK target mutation, often couples with intragenic deletion to result in a constitutively activated form, and ultimately induces primary GBMs 34. It is known that VEGF and its receptors are the critical regulators in glioma angiogenesis 35. In addition, the local degradation of the vascular basement membrane and extracellular matrix play important roles in glioma angiogenesis through promoting he phosphorylation of focal adhesion kinase (FAK) 35. Moreover, EGFR amplification is often associated with *Ink4a* mutation and *TP53* mutation in the EGFR signaling pathway in primary GBMs 36, and the overexpression of the dual mutations of EGFR Ink4a-Arf led to the development of glioma-like lesions 37.

As a major effector of RTK/PI3K pathway, RAS is responsible for the activation of downstream cascades and regulates cellular proliferation and differentiation in various types of cells 38. The RTK/RAS/PI3K pathways promote cell survival. PI3Ks, a family of heterodimeric kinases, have been reported to contain genetic alterations in 88% of GBM cases 39. This pathway is also activated via the loss of negative regulators, such as NF1 and PTEN. As a tumor suppressor gene, *NF1* and its inactivity can lead to the formation of several malignant tumors 40. *NF1* gene inactivation in glial cells and gliomas enhances the activation potential of RAS and its downstream effectors 41. In addition, *NF1* mutant-associated and sporadic astrocytoma, as well as the activations of RAF/MEK/MAPK and PI3K/AKT signaling pathways were determined in glioma patients 16. Moreover, another regulator, PTEN acts as a dual-specificity protein phosphatase and may also impart glioma pathogenesis. PTEN mutations are frequently observed in many malignant cancers, including breast cancer and glioma 42, 43. Moreover, mutated *PTEN* can cause the activation of the AKT signaling pathway, and subsequently regulate the multiple downstream AKT substrates, including FOXO and mTOR 44-46, which are usually downregulated in different types of cancers, including GBMs 47.

### TP53 Pathway

In gliomas, another commonly identified pathway is TP53 signaling pathway (Fig. 3B). As the most common tumor suppressor gene, *TP53* mutation is critical for the progression of glioma 48. TP53 regulates multiple genes that control DNA repair, cell cycle, apoptosis, and progression 49. TP53 pathway also regulates p21 (Waf1/Cip1), which is responsible for silencing cyclin-dependent protein kinases (CDKs) that are essential for the G_1_ to S transition. Li-Fraumeni syndrome, an inherited disorder caused by the presence of a germline mutation in *TP53* gene, predisposes patients to the development of various brain tumors, including astrocytomas 50. In addition, primary *Tp53^-/-^* astrocytes have an increased susceptibility to tumor transformation and growth 51. Interestingly, either *Tp53* homozygous (*Tp53*^-/-^) or heterozygous (*Tp53*^+/-^) mice and zebrafish fail to induce gliomagenesis 9, 52, indicating that the single *Tp53* mutation might be insufficient to initiate gliomagenesis.

### RB1 Pathway

RB1 pathway and its downstream effectors (Fig. 3B), were also genetically and epigenetically regulates in various malignant tumors 53, 54. Specifically, frequent alterations in Rb1 pathway have been observed in 40-70% GBM patients 14, 54. This pathway plays an important role in DNA repair and replication, cellular development, differentiation, migration, mitosis, and apoptosis 55-57. RB1 pathway comprises five protein families: INK4 (p16INK4a, p15INK4b, p18INK4c and p19INK4d), cyclin-dependent protein kinases (CDK6 and CDK4), D-type cyclins (cyclins D3, D2, and D1), RB family proteins (p107, p130 and RB1), and transcription factors (E2F1-8) 58. In this context, p16INK4a and p15INK4b are encoded by the *CDKN2A* and *CDKN2B* genes and bind to CDK4 and CDK6, respectively, to inhibit CDK4/cyclin D1 complex, which results in the inactivation of RB1-mediated G_1_ to S transition 59. Therefore, the inhibition of the components of RB1 pathway might be a promising strategy for the treatment of various malignant cancer types, including astrocytoma, adenocarcinoma, basal cell carcinoma, and gastrointestinal tract endocrine tumor 60-64.

## The progression of glioma models

A suitable animal model can be a useful tool for studying the mechanisms and potential treatments of glioma. Considerable variations exist in brain tumors on the basis of genetic, histological and physiological characteristics, which ultimately contributes to their different malignancies, prognoses, and invasive phenotypes 12, 65, 66. The utility of animal models enables the systematic identification of molecular features, which contribute to the initiation and progression pathways in glioma. The identification of these molecular features could improve the therapeutic strategies of the treatment of glioma. Although the molecular mechanisms of gliomagenesis were widely investigated in cultured cells, the limitations for modeling invasion, angiogenesis and metastasis still exist. An ideal glioma animal model should have several features, including genetic similarity to human glioma, the invasive and angiogenic-like growth, the imitation of the therapeutic response, to allow for more accurately predict the clinical outcome 67. The reviewed literature showed that mice, rats and zebrafish are the most widely used glioma animal models with methods such as chemical induction, xenografting, and bioengineering. To date, several models for brain tumors have been established, as shown in Table 1.

### Chemical Induction

Chemical induction was the first method attempted to induce brain tumors in animal models 67. These induced tumor models did not have a defined genetic background because induction occurred through random chemical substances of bases that gave rise to mispairing or point mutations. Therefore, the location of tumorigenesis, malignancy type, and the incidence conditions were greatly various in each chemical induction study 70, 83, 84. In addition, the tumors induced by the exposure to chemicals is hard to reflect a true representation of the tumorigenesis and tumor progression involved in the patients. Although the application of chemical substances in the generation of glioma models can produce substantial effects and simulate the natural conditions of human glioma, the genetic mechanisms were unclear, and the biological pathways were unspecified 85. Therefore, these critical flaws in traditional glioma models further limit the applied perspective.

### Xenograft Tumor Models

Xenograft tumor models are helpful for studying tumor developmental stages, angiogenesis, invasion, metastasis and the relationship to the host 86, 87. By transplanting CD133-positive and CD133-negative U87 glioma cells in zebrafish, our previous study indicated that glioma stem cells have higher invasion ability than differentiated glioma cells through regulating MMP9 expression 68. A previous report also indicated that the transplantation of U251 cells into zebrafish larvae could evaluate the efficacy and toxicity for anti-cancer molecular compounds 69. Oka et al. showed an adenovirus-mediated REIC/Dkk-3 gene therapy in GL261 glioma cells xenografted mouse model 88. However, although in the ideal state, these xenograft tumor models are technically simple and have lower morbidity rates, these models still do not perfectly represent the actual circumstances of human glioma because of the lack of immunoreactivity in the xenotransplantation model of human glioma cells in immunosuppressed or immunodeficient hosts 89.

### Transgenic Tumor Models

Transgenic models involve molecular-level manipulation of the animal genome in the form of gene editing strategy to induce tumorigenesis 90. Brinster et al. first developed characteristic brain tumors within the choroid plexus via microinjection with the plasmid contains the SV40 early region genes and a metallothionein fusion gene in transgenic mice 91. In addition, previous studies indicated several altered signaling pathways, including ErbB family 92, *EGFR* and *EGFRvIII* signaling 93, were detected in most of human gliomas. Moreover, *TP53* and *PTEN* were mutated in 30-40% glioma patients, suggesting these two tumor suppressor genes are the most frequently altered genes in gliomagenesis. Notably, although these two mutations are commonly found in human gliomas from low-grade to GBMs, previous study showed that the tumors generated in transgenic mice with double mutation of *Pten* and *TP53* were high-grade gliomas (grades III and IV) 36. Further investigation indicated that Tp53, Pten or Rb1 signaling pathway plays different role during gliomagenesis in transgenic mice and zebrafish, and their cooperation could result in high-grade astrocytomas (grades III and IV) in astrocytes and neural precursors in transgenic mice and zebrafish, suggesting that they have different functions for the initiation and progression of gliomagenesis 9, 26.

## Gene editing techniques in studying gliomagenesis

Genome editing nucleases, including ZFNs, TALENS and CRISPR/Cas9, are also emerging tools to detect abnormal gene function in cancer. Gene editing technology has been successfully utilized in mice to study abnormal gene expression during tumorigenesis in liver and lung cancers 94, 95. A previous study established a glioblastoma zebrafish model by TALEN-mediated somatic inactivation of Rb1 using two independent TALEN pairs in zebrafish embryos, which resulted in high-frequency tumor development, mainly in the brain 20. In addition, RCAS/tv-a technology is also commonly used to generate brain tumor models. RCAS-PDGFB-injected SVZ can potentially cause higher grade glioma with a 100% incidence rate and shorter latency. It is noted that the mutations of several tumor suppressor genes, including *Arf*, *Ink4a-Arf* and *Tp53*, has also been determined during tumorigenesis 17. However, although these gene editing technologies could effectively and precisely perform genome editing, their application has been restricted due to factors such as high cost and the difficulty of designing these endonucleases.

In contrast to ZFN and TALEN systems, CRISPR/Cas9 system could efficiently identify any target sequences with convenient design 75, 96. This system based on gRNA, which enables the recognition of the targeted DNA sequences by Cas9 endonuclease, which can subsequently cleave both strands of interest 97-100. In addition, CRISPR/Cas9 is a better technique because of the ease of plasmid construction. The annealing oligonucleotides specify the target site and link into the Cas9 gene-contained vector, which is much easier than the construction of the vector that contains new DNA-binding domain in TALENs or ZFNs system 22. Moreover, the simultaneous generation of multiple mutations is another benefit of CRISPR/Cas9 system, which is very important for establishing disease models because many diseases develop as a result of the abnormal expression of multiple genes rather than dysfunction in a single gene. Thus, CRISPR/Cas9-based glioma models are more suitable for studying the molecular mechanisms of gliomagenesis 21.

Tumorigenesis is often caused by factors such as chromosome aberrations, base deletions or other mutations that ultimately lead to tumor-related genetic modifications. With this novel genomic technology, interactions among molecular mechanisms can be identified in different stages of glioma development. Coupled with the exploitation of relevant signaling pathways, various glioma subtypes can be classified via specific gene expression profiles. CRISPR/Cas9 system is therefore specific, fast, and easy, can lead to stable glioma tumor outcomes and can overcome the limitations of traditional gene editing methods. The CRISPR system can also provide a method to establish tumor models that will be used to study various novel drug target genes and resistance genes for drug treatments 21, 101. Several efforts to establish brain tumor models via CRISPR/Cas9 technology have been successful (Table 1), suggesting that the CRISPR/Cas9-based gene editing technique might open the door to modeling pathological conditions to disclose the mysteries of diverse neurological diseases, including glioma. A previous study indicated the CRISPR/Cas9-mediated inhibition of miR-10b could abolished the neoplastic transformation of normal astrocytes in human glioma cells 102. Luo et al also established a series of transgenic fish lines to tissue-specifically knockout several tumor suppressor genes, including *nf1, tp53, or rb1,* in gliocytes of brain tissue 9. Therefore, this gene editing technology might provide a promising platform for genetically studying tumorigenesis and make it convenient to explore treatment methods for cancer drug targets inspired by CRISPR/Cas9-based genetic screens 103.

## Conclusions and Perspectives

In conclusion, this review highlights the molecular aspects of glioma induction and provides a theoretical basis for establishing a glioma model. Furthermore, the expression and regulation of relevant genes in terms of core pathways have been discussed. Moreover, the method of establishing glioma models using the novel gene editing technology CRISPR/Cas9 and the significance of targeted gene therapy were explained. We believe that the CRISPR/Cas9 technology has a significant potential to enhance the understanding of the mechanisms of gliomagenesis, and enable the development of glioma models and novel and efficient treatment methods.

## Figures and Tables

**Table 1:** Traditional and modern techniques for establishing glioma animal models

Tumor	Method	Genotype/cell line	Animal model	Remarks
GBM	Xenograft	U87MG and U251MG cell lines	zebrafish	A cost-effective approach to investigate glioma invasion, and high-throughput screen or evaluate anti-glioma invasion/metastasis compounds 68, 69.
GBM	Chemical-induced	9L, C6, CNS-1, F98, and RG2	rats	Allows the immune system to interact with the developing tumor, but the genetic mechanism is not clear and the biological process is not stable 70.
GBM	Xenograft	U251MG and U87MG cell lines	mice	Applied widely but is immunosuppressant 71.
GBM^OA^	Transgenic/GFAP promoter	*V12Ha-ras* and *EgfrvIII*	mice	The *EgfrvIII* mutation leads to a constitutively active receptor with impaired internalization, resulting in the activation of pro-invasive signaling pathways 72.
GBM	GFAP-T121	*Pten* ^+/-^	mice	Astrocytoma development is accelerated in a Pten^+/-^ but not a *Tp53^+/-^* background 73.
GBM	Gene knockout	Flox *Nf1* + *Tp53*	mice	A mouse model of astrocytoma with *Tp53* and *Nf1* mutations 74.
GBM^A, AA, OA^	RCAS/tv-a;cre-lox system - deletion of *Pten*	*Pdgfb* and *Ink4a-Arf^-/-^*	mice	The mutations of *Tp53*, *Arf*, or* Ink4a-Arf* can induce higher-grade gliomas 17.
GBM^OA^	Transgenic mice/GFAP	*Pdgfpb* and *Tp53^-/-^*	mice	Tp53 pathway mutations can mediate the transition from low- to high-grade glioma 29.
HGA	GFAP-CreER; PtenloxP/loxP with *Tp53*; *Rb1* double-floxed	*Pten^-/-^, Tp53^-/-^* and *Rb^-/-^*	mice	The combination of the mutations in Pten, Tp53, and Rb1 signaling pathways leads to GBMs in the adult brain of transgenic mice 26.
GBM^OA^	TALEN-mediated somatic inactivation	*rb*-/-	zebrafish	TALEN-mediated the somatic inactivation of Rb1 induces tumorigenesis in genetic mosaic adult zebrafish 20.
GBM^A, AA, OA^	CRISPR/Cas9 system	*Pten*^-/-^, *Tp53*^-/-^ and *Rb^-/-^*	mice	CRISPR/Cas9-based strategies for establishing brain tumor model and investigating pathogenesis of brain tumors 75.
GBM		U251 MG and SNB cell lines		VEGPF was correlative with vascularity and peritumoral edema in CNS tumor 76.
GBM	Gene overexpression	EGFR and PDGFR	human tumor tissue	Overexpression of EGFR and PDGFR in glioblastomas 77.
GBM^A^	Overexpression and deletion	p16, Rb1, CDK4		The expression of cell cycle regulatory genes is rare in secondary glioblastomas 78.
GBM	Overexpression EGFR and deletion p16, p53	EGFR and p16, TP53	human tumor and blood samples	The genetic subtypes were divided into EGFR amplification, *TP53* mutation, and *CDKN2* deletion in GBM patients 79, 80.
GBM	GFAP-Cre; Nf1flox/mut	*Nf1* ^+/-^	mice	mTOR inhibition can suppress Nf1 optic glioma growth 41.
glioma	Transplantation U87 cells	EGFR	zebrafish	The xenografted glioma cells can induce angiogenesis through regulating VEGF expression in zebrafish 81, 82.
GBM^AA^	RCAS/t-va	*Tp53*^-/-^, *Ink4a-Arf*^-/-^	mice	The application of RCAS/t-va technology for establishing glioma mouse model 17.
glioma	CRISPR/Cas9 system	*nf1*^-/-^, *tp53*^-/-^ and *rb1*^-/-^	zebrafish	The crosstalk of three core signaling pathways, including RTK/Ras/PI3K, RB, and TP53 pathways, in gliomagenesis 9.

A, astrocytoma; AA, anaplastic astrocytoma; GS, gliosarcoma; OA, oligoastrocytoma; OG, oligodendroglioma.
